# Intraoperative Injection of Technetium-99m Sulfur Colloid for Sentinel Lymph Node Biopsy in Breast Cancer Patients: A Single Institution Experience

**DOI:** 10.1155/2017/5924802

**Published:** 2017-04-13

**Authors:** Julian Berrocal, Lawrence Saperstein, Baiba Grube, Nina R. Horowitz, Anees B. Chagpar, Brigid K. Killelea, Donald R. Lannin

**Affiliations:** ^1^Department of Surgery, Yale University School of Medicine and Yale Comprehensive Cancer Center, New Haven, CT 06520, USA; ^2^Department of Radiology and Biomedical Imaging, Yale University School of Medicine and Yale Comprehensive Cancer Center, New Haven, CT 06520, USA

## Abstract

*Background*. Most institutions require a patient undergoing sentinel lymph node biopsy to go through nuclear medicine prior to surgery to be injected with radioisotope. This study describes the long-term results using intraoperative injection of radioisotope.* Methods*. Since late 2002, all patients undergoing a sentinel lymph node biopsy at the Yale-New Haven Breast Center underwent intraoperative injection of technetium-99m sulfur colloid. Endpoints included number of sentinel and nonsentinel lymph nodes obtained and number of positive sentinel and nonsentinel lymph nodes.* Results*. At least one sentinel lymph node was obtained in 2,333 out of 2,338 cases of sentinel node biopsy for an identification rate of 99.8%. The median number of sentinel nodes found was 2 and the mean was 2.33 (range: 1–15). There were 512 cases (21.9%) in which a sentinel node was positive for metastatic carcinoma. Of the patients with a positive sentinel lymph node who underwent axillary dissection, there were 242 cases (54.2%) with no additional positive nonsentinel lymph nodes. Advantages of intraoperative injection included increased comfort for the patient and simplification of scheduling. There were no radiation related complications.* Conclusion*. Intraoperative injection of technetium-99m sulfur colloid is convenient, effective, safe, and comfortable for the patient.

## 1. Introduction

Sentinel lymph node biopsy has replaced axillary dissection for axillary staging in clinically node negative breast cancer patients [1]. Although this is considered to be standard of care and there are guidelines and recommendations on how to perform a sentinel lymph node biopsy, there is no standardized protocol for the procedure. There is variability as to which dyes are used, the amount of dye injected, and the timing of injection. When the procedure was first developed in the 1990s, radioisotope was injected into the peritumoral area of the breast and lymphoscintigraphy was performed to evaluate lymphatic drainage into the sentinel nodes over the next hour or two. However, subsequent studies showed that intradermal and periareolar injection of the isotope resulted in faster uptake into the nodes and yielded a higher identification rate [2–6]. Furthermore, it was found that routine lymphoscintigraphy was not necessary as accurate identification of sentinel nodes was possible without the preoperative images [7]. This made intraoperative injection a particularly feasible approach and several small series reported excellent results [8–10]. These series showed clearly that intraoperative injection resulted in decreased patient discomfort and markedly improved efficiency of scheduling for both the patient and surgeon. Despite clear advantages to intraoperative injection, many institutions still require patients to go through nuclear medicine for injection prior to surgery. At Yale, we have used intraoperative injection of technetium-99m sulfur colloid for all sentinel node biopsies since late 2002. The aim of this study is to describe our long-term experience with the technique in more than 2,300 breast cancer patients.

## 2. Methods

### 2.1. Sentinel Lymph Node Procedure

**Figure 1:**
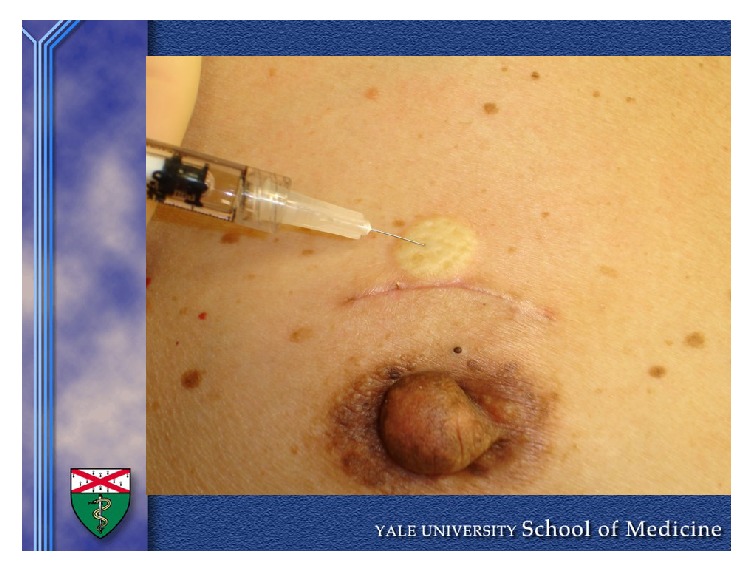
Intradermal injection of Tc-99m above excisional biopsy scar.

On the day of surgery, a nuclear medicine technologist delivers a lead case to the operating room containing 0.25–0.5 mCi of technetium-99m sulfur colloid in a 0.4 mL volume. After induction of general anesthesia, the radioactive tracer is infiltrated intradermally as a skin wheal directly over the breast cancer site or injected into the subdermal plexus of the areola. In rare circumstances in which there is a scar between the areola and the axilla, the radioisotope is injected lateral to the scar so as to avoid interference from scar tissue (see Figure 1). The surgeon then preps and drapes the patient in the usual manner. If dual tracer is desired by the surgeon, isosulfan blue or diluted methylene blue dye is also injected either peritumorally or into the subareolar plexus. About 10 to 15 minutes after injection of isotope, a hot spot can be identified in the axilla with a gamma probe and a small incision made over it. After entering the clavipectoral fascia, the gamma probe is used to identify the hottest node and any other hot nodes demonstrating greater than 10% of counts of the hottest node or any node that is blue or found to feel suspicious. The sentinel lymph nodes are removed and sent to pathology for frozen section or for permanent section based on surgeon preference.

### 2.2. Nuclear Medicine Protocol for Intraoperative Technetium Injection

The licensed nuclear radiology attending physician authorizes release of technetium for intraoperative injection. The surgeons have received instruction on how to properly handle the isotope. For each case where SLNB is scheduled, nuclear medicine staff delivers Tc-99m sulfur colloid in a lead box from the department of nuclear medicine to the appropriate operating room. The surgeon then carefully injects the Tc-99m sulfur colloid while wearing gloves and eye protection. The empty syringe, needle, and any gauze or alcohol wipes that come into contact with the isotope are immediately placed back into the lead box, which is then taken back to the nuclear medicine department for proper disposal. A radiation safety officer is available at all times for questions or to respond to spills. To date, there have been no radioactive spills in the operating room or any inappropriate disposal of radioactive waste. Surgeons and OR staff are not required to wear radioactive monitoring badges given the limited exposure.

### 2.3. Data Collection and Statistics

The Breast Center maintains a prospectively collected database which contains data on all patients undergoing oncologic breast surgery. After approval by the Yale University Human Investigations Committee (HIC), patient deidentified data was abstracted from this database on all patients undergoing a sentinel lymph node biopsy for breast cancer between 2003 and 2014. Demographic data collected included patient age, ethnicity, cancer histology, molecular subtype, TNM stage, and breast surgical procedure, and endpoints collected included the number of sentinel lymph nodes obtained, number of positive sentinel lymph nodes, number of nonsentinel lymph nodes obtained, and number of positive nonsentinel lymph nodes. Because the data was merely descriptive, no statistical comparisons were needed.

## 3. Results

There were 2,333 patients in the database who underwent a sentinel lymph node biopsy. All patients had radioisotope injection of technetium-99m sulfur colloid and approximately 56% of cases had dual tracer with both the radioisotope and blue dye. During the early part of the time period, there were 5 additional cases where a sentinel node biopsy was attempted but no node was found, for an identification rate of 99.8%. All of the five cases where a node was not found had an extensive scar between the injection site and the axilla. This leads to our current practice of injecting the isotope in a location to avoid interference with a surgical scar. We also had a number of cases where a second sentinel node biopsy was performed after a previous sentinel node biopsy, but these cases were excluded from this analysis.

### 3.1. Patient Demographics

The patient demographics for all 2,333 patients are shown in Table 1. The majority of patients were Caucasian (80.3%), with Blacks representing 9.5% of the study population. The average patient age was 56.9 years, with 77.5% of the patient population in the age range of 41–70 years. Most cancers were infiltrating ductal carcinoma (67.4%), with ductal carcinoma in situ (DCIS) and infiltrating lobular carcinoma representing 10.8% and 10.4%, respectively. The molecular subtype of tumors most represented in the study population was ER/PR positive and Her-2 negative (64%). Over 94% of the cancers in this study had an early T stage (DCIS or stage I/II) and the majority (47.6%) were moderately differentiated. Partial mastectomy was more commonly performed than total mastectomy (54.7% versus 45.3%). This rate of mastectomy is higher than our overall rate because our practice has been to perform sentinel node biopsy on patients with DCIS who undergo mastectomy but not on most DCIS patients undergoing lumpectomy. Thus, DCIS patients receiving lumpectomy would not be included in this analysis.

### 3.2. Sentinel Lymph Nodes

The median number of sentinel nodes obtained was 2 and the mean number was 2.33 with a range of 1–15 nodes. About a third of cases had only one sentinel lymph node removed, a third had 2, and a third had 3 or more (Table 2). Of all the sentinel lymph nodes obtained, only 512 cases (21.9%) were positive for metastatic carcinoma as shown in Table 3. In the subset of patients with invasive carcinoma, 24.6% had at least one positive sentinel lymph node (Table 4). However, of the patients with in situ carcinoma, there was only one case which had a positive sentinel lymph node. There were other cases with a positive sentinel node where the preoperative diagnosis based on the needle biopsy was DCIS, but all of these cases except for one were upgraded to invasive breast cancer on the final pathology.

### 3.3. Nonsentinel Lymph Nodes

Table 5 shows the number of nonsentinel lymph nodes obtained following a positive sentinel lymph node biopsy. Until 2011, almost all of our patients who had a positive sentinel node underwent axillary dissection. However, following the publication of the ACOSOG Z11 trial [11], there were 87 cases (16.3% of the entire group) in which no further lymph nodes were excised. Of the patients with a positive sentinel lymph node biopsy who underwent a completion axillary lymph node dissection, 242 cases (54.2%) had no additional positive lymph nodes as shown in Table 6.

## 4. Discussion

Although historically an axillary lymph node dissection was performed to stage the axilla, compared to sentinel lymph node biopsy, there was increased morbidity including lymphedema, nerve disruption, chronic shoulder pain, weakness, and joint dysfunction. As a result, sentinel lymph node biopsy has become standard of care to allow for proper axillary lymph node staging [1, 12]. This is usually performed with radioactive Tc-99m labeled sulfur colloid [13], vital blue dye such as isosulfan blue or methylene blue [14], or the combination [15, 16]. Studies performed during the learning curve for the procedure usually show a higher identification rate using both blue dye and radioisotope in combination [16, 17]. However, there are occasional allergic reactions to the blue dyes, including anaphylactic reactions, urticaria, rash, blue hives, and pruritus associated with isosulfan blue dye and skin necrosis associated with nondiluted methylene blue. Many surgeons have found that, with extensive experience, the blue dye is not always necessary and can be used selectively in the rare cases where radioactivity is not detected in a node. The results of our current study show that it is possible to get essentially a 100% identification rate either with the combination of isotope and blue dye or with isotope alone.

With the speed of lymph node localization provided by intradermal and subareolar injection [2–6] and the realization that lymphoscintigraphy images are not needed [7], there is no reason not to inject the isotope in the operating room after induction of anesthesia. Several small series have shown that this provides excellent results. A study by Layeeque et al. [9] demonstrated successful use of intraoperative subareolar technetium-99m sulfur colloid and blue dye. Ninety-six sentinel lymph node biopsy procedures were performed in 88 breast cancer patients, and 97% percent of the cases demonstrated a “hot” sentinel lymph node. Similar to our experience, the three percent of patients that did not have successful localization had a history of prior surgery between the injection site and the axilla. Zogakis et al. [10] used intraoperative subareolar injection of Tc-99m labeled sulfur colloid along with blue dye in 122 patients and found a sentinel node in 99.2%. Dauphine et al. [8] injected 100 consecutive patients intraoperatively with Tc-99m labeled sulfur colloid immediately after induction of anesthesia and compared this group to the previous 100 patients who had been injected preoperatively. The sentinel node identification rate was 100% in the intraoperative group compared to 96% in the preoperative group; there was no difference in the number of nodes found or the percent of nodes that were positive for metastases. The current report of 2,333 patients injected intraoperatively represents the largest series in the literature.

The benefits of intraoperative injection in our experience include both patient and surgeon related factors as outlined in Box 1. From a patient standpoint, pain at the time of radioisotope injection was one of the most common complaints with preoperative injection. Injected local anesthesia was not given as it could impact the uptake of the radioisotope within the lymphatics. From a logistical standpoint, patients do not have to travel to other departments on the day of surgery if an intraoperative injection of radioisotope is performed. From a surgical scheduling standpoint, intraoperative injection allows greater flexibility as a case can start early in the morning, there does not need to be coordinated scheduling between surgery and nuclear medicine, and there are never any surgical delays due to patients being held longer than expected in nuclear medicine.

It is important to point out that intraoperative injection requires collaboration between the surgeons and nuclear radiologists. Ultimately the nuclear radiologist bears the responsibility for the safe use of the isotopes. From a radiation safety standpoint, Miner et al. [18] demonstrated that the procedure is safe. A surgeon could safely perform 1,000 procedures per year without surpassing the OSHA defined safety limit. Stratmann et al. [19] conducted a study to investigate radiation exposure to the surgeon, scrub nurse, pathologist, and OR equipment to determine safety of sentinel lymph node biopsy with radioisotope. They concluded that a primary surgeon could perform 2,190 hours, a scrub nurse 33,333 hours, and a pathologist 14,705 hours of procedural work with radioisotope annually before surpassing radiation safety limits set forth by OSHA. Furthermore, operative instruments, pathology slides, and cryostat machines require no special handling following a sentinel lymph node biopsy with radioisotope. In the present study, all radioactive material was placed into a lead case during delivery to the operating suite and all material which contacted the radioactive isotope was returned to nuclear medicine within the lead case. No complications occurred as a result of the radioactive isotope.

We are not advocating that intraoperative injection should become a standard of care that is used everywhere. Perhaps at institutions with a large number of low volume breast surgeons, intraoperative injection may not be feasible. Each institution should decide what works best for its particular situation. However, at Yale, with a small number of high volume breast surgeons, intraoperative injection works very well.

## 5. Conclusion

In our long-term, prospective experience, intraoperative injection of the radioisotope, technetium-99m sulfur colloid is convenient, effective, safe, and comfortable for the patient. The sentinel lymph node detection rate was essentially 100%.

## Figures and Tables

**Box 1:**
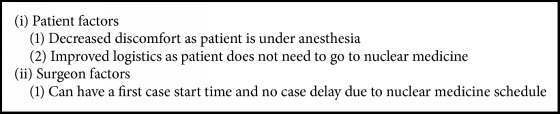
Advantages of intraoperative radioisotope injection.

**Table 1:** Demographic data.

	Frequency	Percent
*Ethnicity*		
Caucasian	1,874	80.3
Black	222	9.5
Hispanic	113	4.8
Asian	66	2.8
Other	58	2.5
*Age (years)*		
<30	22	0.9
31–40	164	7
41–50	608	26.1
51–60	649	27.8
61–70	553	23.7
>71	337	14.4
*Tumor Histology*		
Infiltrating ductal	1,573	67.4
Infiltrating lobular	242	10.4
Mixed ductal and lobular	137	5.9
Ductal carcinoma in situ	253	10.8
Other	128	5.5
*Molecular subtype*		
ER/PR positive, Her-2 negative	1,494	64
ER/PR positive, Her-2 positive	160	6.9
ER/PR negative, Her-2 positive	102	4.4
ER/PR negative, Her-2 negative	243	10.4
*T stage*		
0	253	10.8
1	1,366	58.6
2	592	25.4
3	111	4.8
4	11	0.5
*Tumor grade*		
Well differentiated	499	21.4
Moderately differentiated	1,110	47.6
Poorly differentiated	557	23.9
*Surgery*		
Partial mastectomy	1,277	54.7
Mastectomy (including bilateral, simple, and modified radical)	1,056	45.3

**Table 2:** Sentinel lymph nodes obtained.

	Frequency	Percent
1	779	33.4
2	754	32.3
≥3	800	34.3
Total	2,333	100

**Table 3:** Positive sentinel lymph nodes.

	Frequency	Percent
0	1,821	78.1
1	365	15.6
2	101	4.3
≥3	46	2
Total	2,333	100

**Table 4:** Positive sentinel lymph nodes by histology.

	Invasive carcinoma	Ductal carcinoma in situ
0	1,569 (75.4%)	252 (99.6%)
1	365 (17.5%)	0 (0%)
2	100 (4.8%)	1 (0.4%)
≥3	46 (2.2%)	0 (0%)
Total	2,080	253

**Table 5:** Nonsentinel lymph nodes obtained in sentinel lymph node positive patients.

Nonsentinel lymph nodes	Frequency	Percent
0	87	16.3
1–10	203	38
11–20	199	37.4
21–30	68	7.8
31–39	4	0.8

**Table 6:** Positive nonsentinel lymph nodes following positive sentinel lymph node biopsy.

Positive nonsentinel lymph nodes	Cases with a positive sentinel lymph node	Percent
0	242	54.2
1	65	14.5
2	42	9.4
3	22	4.9
4	17	3.8
5–10	38	8.4
11–20	15	3.2
21–29	6	1.2
